# SLEEP CONCORDANCE AMONG CARE DYADS OVER TIME: DOES IT VARY BY CAREGIVING CONTEXTS?

**DOI:** 10.1093/geroni/igae098.1830

**Published:** 2024-12-31

**Authors:** Dexia Kong, Chihua Li, Xiaoling Xiang, Peiyi Lu

**Affiliations:** The Chinese University of Hong Kong, Hong Kong, China (People’s Republic); University of Michigan, Ann Arbor, Michigan, United States; University of Michigan, Ann Arbor, Michigan, United States; The University of Hong Kong, Hong Kong, China (People’s Republic)

## Abstract

The well-being of care recipients (CR) and informal caregivers (CG) is intricately interconnected. However, research on sleep concordance among caregiving dyads is scarce. Leveraging longitudinal dyadic data and a cross-lagged panel design, this study aims to investigate whether: 1) sleep patterns are concordant among caregiving dyads over time; and 2) the extent of sleep concordance differs by caregiving contexts (i.e., dementia caregiving versus non-dementia caregiving) or by living arrangements (i.e., living together versus living apart). Longitudinal data from the National Health and Aging Trends Study (NHATS) and the National Study of Caregiving (NSOC) were included (N = 2,204 care dyads). Cross-lagged panel models and multiple group analysis were used. Sleep problems of CR and CG in 2015 were significantly associated with their own sleep problems in 2017. The partner effects were statistically insignificant (P>0.05). However, CR sleep problems in 2015 were associated with more CG sleep problems in 2015 (β=0.141, 95% CI=0.015, 0.268), which were further linked to more CG sleep problems in 2017 (β=0.504, 95% CI=0.431, 0.577). Furthermore, these relationship patterns remained significant among dementia care or co-residing dyads, but not among non-dementia or living apart care dyads. CR sleep problems could affect CG sleep synchronously, potentially leading to long-term deterioration of CG sleep. This can be attributed to the cumulative physical exhaustion and psychological strain associated with caregiving. Our findings highlight the importance of early intervention in mitigating CR sleep problems and targeted assistance to CG of persons with dementia and those co-residing with their CR.

